# Antimicrobial activity of mesenchymal stem cells against *Staphylococcus aureus*

**DOI:** 10.1186/s13287-020-01807-3

**Published:** 2020-07-17

**Authors:** Haruyo Yagi, Antonia F. Chen, David Hirsch, Adam C. Rothenberg, Jian Tan, Peter G. Alexander, Rocky S. Tuan

**Affiliations:** 1 Department of Orthopaedic Surgery, Center for Cellular and Molecular Engineering, University of Pittsburgh School of Medicine, 450 Technology Drive, Bridgeside Point II, Pittsburgh, PA 15219 USA; 2 Present address: Department of Orthopaedic Surgery, Brigham and Women’s Hospital, Harvard Medical School, Boston, MA USA; 3 Present addresses: EvergreenHealth Orthopedic & Sports Care, Kirkland, WA USA; 4 Present address The Chinese University of Hong Kong, Institute for Tissue Engineering and Regenerative Medicine, Shatin, Hong Kong, SAR China

**Keywords:** Adipose-derived mesenchymal stem cells, Antimicrobial effect, LL-37, Vitamin D_3_

## Abstract

**Introduction:**

There have been limited advances in the treatment of bone and joint infections, which currently involves a combination of surgery and antibiotic administration. There is a timely need in orthopedics to develop more effective and less invasive forms of antimicrobial prophylaxis and treatment. The antibacterial effect of adult tissue-derived mesenchymal stem cells (MSCs) has recently been investigated against *Escherichia coli* and *Staphylococcus aureus*. The main mechanism of action is postulated to be via MSC production of the cationic antimicrobial peptide, LL-37.

**Methods:**

This study examines the antimicrobial activity of adipose-derived human MSCs (ASCs) on *S. aureus*, specifically examining the role of LL-37 and regulation of its expression. Bacteria colony-forming unit (CFU) assay was used to assess antimicrobial activity.

**Results:**

Our results showed that the ASC-conditioned medium significantly inhibited the growth of *S. aureus* under standard culture conditions with or without the continued presence of ASCs. Also, the treatment of ASCs with 1,25-dihydroxy vitamin D_3_ elevated LL-37 expression and enhanced their antimicrobial activity. In support, treatment with the vitamin D receptor inhibitor, GW0742, blocked the antimicrobial activity of ASCs.

**Conclusion:**

Our findings clearly demonstrate the antimicrobial activity of adult ASCs against *S. aureus* and implicate a key regulatory role for vitamin D. Further testing in in vivo models is being pursued to assess the potential application of ASCs as a biocompatible, adjunct treatment for musculoskeletal infections.

## Introduction

Osteomyelitis (OM), or infection in the bone, affects approximately 15% of patients with extremity injuries, of which about 17% experience recurrent infection [1]. Common wound pathogens include *Escherichia coli* (*E. coli*), *Staphylococcus aureus* (*S. aureus*), and methicillin-resistant *S. aureus* (MRSA), as well as multidrug-resistant bacteria, including *Acinetobacter baumannii*, *Pseudomonas aeruginosa*, and extended-spectrum β-lactamase-producing *Klebsiella* species [1]. Although initial infections are often complicated by Gram-positive pathogens, many of the late relapses involve both Gram-negative and Gram-positive bacteria, commonly methicillin-sensitive *S. aureus* (MSSA) and MRSA [1].

Bone infection, at sites of relatively poor vascularity, can be difficult to treat, often requiring prolonged courses of antimicrobial therapy in association with surgical drainage or debridement [2–4]. The delayed or ineffective treatment causes significant morbidity in terms of pain, loss of function, and the need for further surgery and antibiotics [2, 3]. Antimicrobial activity through systemic and possibly local delivery is often needed to prevent subsequent infections. The first line of treatment consists of wound irrigation and debridement of devitalized and contaminated tissue. Repeat surgical debridement and use of intravenous systemic antibiotics are almost always required. Long-term treatment with systemic antibiotics increases bacterial resistance through the selection of resistant strains. Bone penetration of many antibiotics has been studied [5–10], but the interpretation of results is difficult as the methodologies have not been standardized, and therefore, results have varied, but commonly used antibiotics such as vancomycin have poor bone penetration [2, 7, 11]. Recent attempts at local antibiotic delivery through an indwelling catheter in joint infections have only marginally improved outcomes [12–14]. Since these methods were introduced in the 1970s, there has been limited advancement in treatment. Local antibiotic delivery in the form of antibiotic powder, irrigation solutions, bone cement, collagen sponges, pastes, and bone graft have complications including seromas, ototoxicity, nephrotoxicity, and compromised osteoblast activity [15]. There is a need in orthopedics to provide a longer-lasting, more effective, and less invasive forms of antimicrobial prophylaxis and treatment for OM. Our approach is to augment the patient’s own immune system to combat infection.

Mesenchymal stem cells (MSCs) can be easily isolated from various adult tissue sources such as the bone marrow (BM), adipose, bone, and muscle and retain their differentiation potential during in vitro culture expansion and manipulation [16–18]. Although BM-MSCs have been shown to exhibit robust differentiation potential, only a limited number of MSCs can be obtained from a single BM aspirate, and culture expansion in vitro is required, which leads to a loss of potency [19]. Recent studies have focused on adipose-derived stromal/stem cells (ASCs) as an alternative autologous MSC source that is free of the controversy and limitations of embryonic stem cells and BM-MSCs [20–23]. ASCs possess a substantial potential for regenerative medicine, and adipose tissues have been shown to contain 100- to 2500-fold greater numbers of stem cells per volume than BM aspirates [24–26]. Importantly, these ASCs can be quickly isolated using differential centrifugation in the operating room, making ASCs a viable candidate for use in any point-of-care procedure. In addition, like MSCs, ASCs secrete a multitude of therapeutic growth factors and cytokines that are anti-inflammatory and immunomodulatory [27–33].

The antibacterial effect of MSCs has been investigated in other fields, including pulmonary medicine [34–41]. A recent study demonstrated that MSCs possess direct antimicrobial activity in vitro against * E. coli* and * S. aureus* [35]. These human MSCs were also effective in vivo in reducing the bacterial count in a mouse model of *E. coli*-mediated pneumonia [35]. Our own recent findings showed that the addition of MSCs effectively suppressed the growth of *E. coli* in an in vitro simulated synovial environment [42]. The main mechanism of action is reported to be via LL-37, a cationic antimicrobial peptide expressed by immune cells and other epithelial cells in the body [35]. LL-37 is a cleavage product of the cathelicidin, hCAP-18, the human member of the cathelicidin family of antimicrobial proteins originally found in the peroxidase-negative granules of neutrophils [43]. LL-37 also has other antimicrobial properties, including antifungal [44] and antiviral activities [45], and the inhibition of biofilm formation [46, 47]. LL-37 is functionally linked to the modulation of Toll-like receptors (TLRs) and can be induced by stimulation through TLR2, TLR4, and TLR9 [48, 49]. Recent studies also showed that TLRs are stimulated by the active form of vitamin D_3_ (1,25-dihydroxy vitamin D_3_, 1,25(OH)_2_D_3_) [50], reportedly resulting in higher LL-37 expression [48, 51]. Therefore, in this study, 1,25(OH)_2_D_3_ was used to activate ASCs. We hypothesized an enhancement of the antibacterial activity of ASCs upon activation by 1,25(OH)_2_D_3_. The effect on ASCs was examined by analyzing the expression and regulation of LL-37, the candidate antimicrobial product responsible for the bactericidal activity of MSCs. Our results showed that ASCs exhibited significant antimicrobial activity, which was enhanced by 1,25(OH)_2_D_3_, mediated via vitamin D receptor signaling.

## Materials and methods

### Chemicals and reagents

Dulbecco’s modified Eagle’s medium (DMEM), α-Minimum Essential Medium (α-MEM), antibiotic-antimycotic (anti-anti), fetal bovine serum (FBS), and phosphate-buffered saline (PBS) were purchased from the Life Technologies (Grand Island, NY). OxPAPC, CLI-095, and monophosphoryl lipid A (MPLA) were purchased from the Invivogen (San Diego, CA). Fibroblast growth factor-2 (FGF-2) was obtained from the R&D System (Minneapolis, MN). Tryptic soy broth was purchased from the Acumedia (Lansing, MI). Agar was obtained from the Fisher Scientific (Pittsburgh, PA). Human LL-37 ELISA kit was purchased from the Hycult Biotech (Plymouth Meeting, PA). LL-37 peptide was purchased from the AnaSpec (Fremont, CA). GW0742, trypsin IIS, and ampicillin (Amp) were purchased from the Sigma-Aldrich (St. Louis, MO). Collagenase type I was purchased from the Worthington (Lakewood, NJ). 1,25-dihydroxy vitamin D_3_ was purchased from the Cayman Chemical (Ann Arbor, MI).

### Preparation of human BM-MSCs and ASCs

Human BM was harvested from femoral heads of patients (17 to 60 years of age, 1 female and 2 males) undergoing total hip arthroplasty, according to an Institutional Review Board (IRB) exempted approval protocol (University of Washington School of Medicine). MSCs were obtained as tissue culture plastic adherent cell populations [52]. The trabecular bone was cored out using a curette or rongeur, and the bone marrow was flushed out with rinsing medium (α-MEM, 1% anti-anti) using 18-gauge hypodermic needles. After mincing with scissors, the bone chips were flushed, and the flushed medium was passed through 40-μm mesh filters to remove debris, and cells pelleted by centrifugation for 5 min at 300×*g*. Cell pellets were washed twice with rinsing medium and resuspended in MSC growth medium (GM, α-MEM) containing 10% FBS, 1% anti-anti (Invitrogen, Carlsbad, CA), and 1 ng/mL FGF-2 and plated into two 150 cm^2^ tissue culture flasks. On day 4, cells were washed with PBS and fresh GM was added. GM was changed every 3–4 days. Once 70 ~ 80% confluence was reached, cells were removed with 0.25% trypsin containing 1 mM EDTA and passaged by re-plating. Populations of MSCs isolated from each patient were routinely validated as capable of undergoing osteogenic, adipogenic, and chondrogenic differentiation using established protocols (data not shown). All experiments were performed with passage 1–4 cells.

Human infrapatellar fat pad (IPFP) was harvested from knees of patients (52 to 88 years of age, 10 females and 2 males) undergoing total knee arthroplasty, according to an IRB exempted approval protocol (University of Washington School of Medicine). ASCs were obtained as tissue culture plastic adherent cell populations. IPFP was dissected from the knee and placed in a Petri dish with the Hank’s Balanced Salt Solution (HBSS, Hyclone), minced into small pieces (~ 1 mm^3^), and any obvious fibrous tissue was removed. Collagenase type I (10 mg/cc wet tissue) and trypsin IIS (10 mg/cc wet tissue) were added to the conical tube containing the fat (up to 10 cc wet tissue). The fat was agitated continuously at 180 rpm, 37 °C for 3 h, and tissue digestion was stopped by adding growth medium (with 10% serum). The tissue digest was then filtered through 100 μm cell strainer, centrifuged at 1500 rpm (400 g) for 5 min, and the pellet resuspended in 25 mL growth medium. The tissue digest was filtered again through 100 μm cell strainer and centrifuged at 1500 rpm (400 g) for 5 min. The cell pellets were resuspended in ASC growth medium (a-MEM) containing 10% FBS, 1% antibiotic-antifungal mix (Invitrogen, Carlsbad, CA), and 1 ng/mL FGF-2 and counted and distributed to T-150 tissue culture flasks at ~ 1 million cells/flask. Populations of ASCs isolated from each patient were routinely validated as capable of undergoing osteogenic, adipogenic, and chondrogenic differentiation using established protocols (data not shown). All experiments were performed with cells at passage 2 to 4.

### Bacteria

*Staphylococcus aureus*, the most common Gram-positive organism present in osteomyelitis, was chosen for the testing of antimicrobial activity. *S. aureus* (ATCC 25923) was grown in Tryptic Soy Broth (TSB) medium for 16 h at 37 °C, and cultures were adjusted to 3 × 10^4^ colony-forming units (CFU)/mL on the basis of optical absorbance (A_600_ 1.0 equivalent to 6.6 × 10^8^ CFU/mL). Amp was used as the antibiotic against *S. aureus.*

### Assay for antibacterial activity

Human BM-MSCs or human ASCs expanded in growth medium were placed in fresh, antibiotic-free growth medium for 24 h, after which cells were rinsed with physiological saline, trypsinized, and pelleted by centrifugation at 1100 rpm for 5 min. The pelleted cells were then resuspended in growth medium without antibiotics, and seeded in non-tissue culture-treated 6-well plates and cultured for another day. The overall experimental scheme is shown in Fig. 2. The following groups were analyzed: (1) group 1—no bacteria control (growth medium without antibiotic); (2) group 2—bacteria control (growth medium without antibiotic, inoculated with bacteria); (3) group 3—cells in conditioned medium, inoculated with bacteria; (4) group 4—cells in control medium, inoculated with bacteria; and (5) group 5—conditioned medium only, inoculated with bacteria. To assay for antibacterial activity, 2 mL/well aliquots of medium were taken from all wells in the 6-well plates and, except group 1 (no bacteria control), were then inoculated with 3 × 10^3^*S. aureus* ± 12.5 μg/mL of Amp. All samples were then placed for 1 h at 37 °C in 5% CO_2_ incubator. At the end of incubation, a 100-μL aliquot of the contents of each well was plated on TSB agar. After 16 to 18 h incubation at 37 °C, bacterial colonies were counted manually using a digital colony counter pen (Fisher Scientific) and the results compared against the bacteria control (group 2) and expressed as percentage values.

### Synovial fluid

To assess the antimicrobial activity of BM-MSCs/ASCs in a simulated synovial environment, synovial fluid was introduced into the assay mix. Aseptic synovial fluid was collected from one patient undergoing routine elective total knee arthroplasty who showed no clinical sign of infection, under an exempted IRB protocol (University of Pittsburgh, PRO14020504). BM-MSCs/ASCs were first cultured in antibiotic-free growth medium for 24 h, after which the cells with the respective cell-conditioned medium were replated (1 × 10^5^ cells/well) into non-tissue culture coated 6-well plates, and the control consisted of cell-free growth medium that was also pre-incubated for 24 h. An aliquot (200 μL/well) of synovial fluid was added to the 400 μL cultures to simulate the synovial environment. To assay for antibacterial activity, an aliquot of *S. aureus* (3.3 × 10^3^ CFU in 25 μL) was inoculated into each well, followed by incubation for 1 h at 37 °C in 5% CO_2_ incubator, and then 30 μL of the inoculated medium was used for colony-count plating (in triplicates) as described above.

### Treatment with 1,25-dihydroxy vitamin D_3_

Twenty-four hours before the experiment, the culture medium of cultured cells was replaced with a fresh antibiotic-free growth medium. On the day of the experiment, cells were rinsed with physiological saline and released by trypsinization, pelleted by centrifugation at 1100 rpm for 5 min, and were resuspended in growth medium without antibiotics. Finally, the cells were replated in tissue culture-treated 6-well plates (0.5 × 10^5^ cells/2 mL/well). Cells were then kept at 37 °C in 5% CO_2_ incubator for 2 h, after which the cultures were treated with 1,25(OH)_2_D_3_ (0 to 100 nM) for varying time periods (ASCs: 15 min, 30 min, 1 h, 2 h, 4 h, and 6 h, BM-MSCs: 30 min, 1 h, and 6 h; 1,25(OH)_2_D_3_ 100 nM = approximately 40 ng/mL). Unless otherwise specified, an antibiotic-free culture medium was used throughout the entire study. At the end of the desired exposure time, all media were removed and saved for CFU assay. Briefly, 1 mL/well aliquots of medium were inoculated with 1.5 × 10^3^*S. aureus*. All samples were then placed for 1 h at 37 °C in 5% CO_2_ incubator. At the end of incubation, a 50 μL aliquot of the contents of each well was plated on TSB agar.

### qRT-PCR assay

The wells were first washed twice with 2 mL of PBS, then extracted with 350 μL of RLT Plus buffer (Qiagen RNeasy Kit) for 10 min. Afterwards, RNA was isolated with the RNeasy kit and converted to cDNA by using First-Strand IV Synthesis Supermix (ThermoFisher) and analyzed by qRT-PCR for LL-37. Primers used here included (1) LL-37 (5′-region of transcript) - forward, 5′-GAA GGC TCC TGG TTG GG-3′; reverse, 5′-TCT GCC TCC CTC TAG CC-3′; (2) LL-37 (middle of transcript): forward, 5′-ATC ATT GCC CAG GTC CTC AG-3′; reverse: 5′-GTC CCC ATA CAC CGC TTC AC-3′; and (3) GAPDH: forward, 5′-AGC CAC ATC GCT CAG ACA C-3′; reverse, 5′- GCC CAA TAC GAC CAA ATC C-3′.

### Treatment with antagonists of 1,25(OH)_2_D_3_ receptor and TLR receptors

Twenty-four hours prior to the experiment, cultures of human ASCs maintained in growth medium were changed to the fresh antibiotic-free growth medium. On the day of the experiment, cells were rinsed with PBS, released by trypsinization, and pelleted by centrifugation at 1100 rpm for 5 min. The pelleted cells were resuspended in growth medium without antibiotics. Finally, the cells were replated in non-tissue culture-treated 6-well plates (0.5 × 10^5^ cells/2 mL/well) and treated with the following: (1) TLR agonists–monophosphoryl lipid A (MPLA; 12.5 μg/mL), TLR4 antagonist CLI-095 (2.5 μM), or TLR2/4 antagonist OXPAPC (25 μM) for 1 day; or (2) vitamin D receptor inhibitor GW0742 (12.5 μM), or 1,25(OH)_2_D_3_ (100 nM) for 1 day. To test for antimicrobial activity, 1 mL of conditioned medium was inoculated with 1.5 × 10^3^*S. aureus* ± 25 μg/mL of ampicillin and incubated for 1 h at 37 °C in 5% CO_2_ incubator. An ASC-free control was processed under the same conditions. At the end of the incubation period, a 50-μL aliquot of the medium from each well (bacteria and medium) was plated on TSB agar for CFU counting as described above. Unless otherwise specified, an antibiotic-free culture medium was used throughout the entire study.

### Statistical analysis

Significant differences (*p* < 0.05) were assessed with two-tailed Student’s *t* test for two-group comparisons using Microsoft Excel, and *p* values are indicated in the figures.

## Results

### BM-MSCs and ASCs both inhibited bacterial growth in synovial fluid

**Fig. 1:**
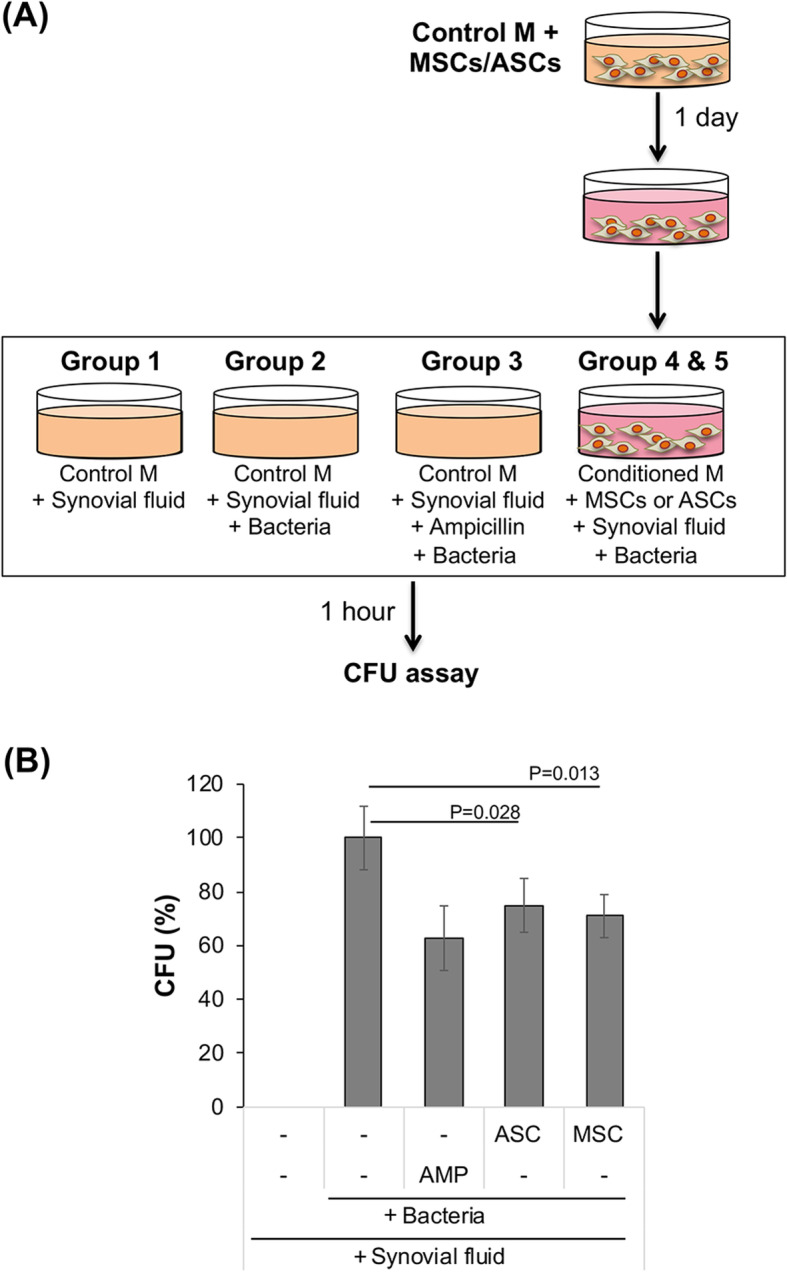
BM-MSCs/ASCs suppressed bacterial growth in the presence of synovial fluid. **a** Experimental scheme. For details see the “Materials and methods.” M, medium. **b** Antibacterial activity of BM-MSCs/ASCs in the presence of synovial fluid. Uninfected synovial fluid was added to the control medium to simulate a synovial environment. *S. aureus* (3.3 × 10^3^ CFU) was then added, with or without BM-MSCs/ASCs, and the mixture was incubated for 1 h at 37 °C under 5% CO_2_, and an aliquot was removed to test for bacterial CFU. In some samples, ampicillin (AMP; 12.5 μg/mL) was added just before bacterial inoculation. After the plated dishes were incubated for 16 h at 37 °C, colonies were counted. CFU results (mean ± SD) are from 6 technical replicates and expressed as percentages of the bacteria control

As osteomyelitis commonly occurs in relationship to joint infection, we simulated the synovial environment in our in vitro assay by the addition of uninfected synovial fluid in the assay mixture, followed by bacterial inoculation to model joint infection for all experimental groups (Fig. 1a). The results in Fig. 1b showed that no bacterial growth was observed in the negative control group consisting of culture medium alone. In contrast, the positive bacteria control group showed 148 ± 18 CFU/plate (set as 100%), which was inhibited with ampicillin. When BM-MSCs were added to *S. aureus*, bacterial growth was significantly inhibited (71 ± 8%, *p* = 0.013 versus bacteria control). Similarly, when ASCs were added to *S. aureus*, bacterial growth was also significantly inhibited (75 ± 10%, *p* = 0.028 versus bacteria control). No significant differences were observed between ASCs and BM-MSCs.

### Antibacterial activity of BM-MSCs and ASCs

**Fig. 2:**
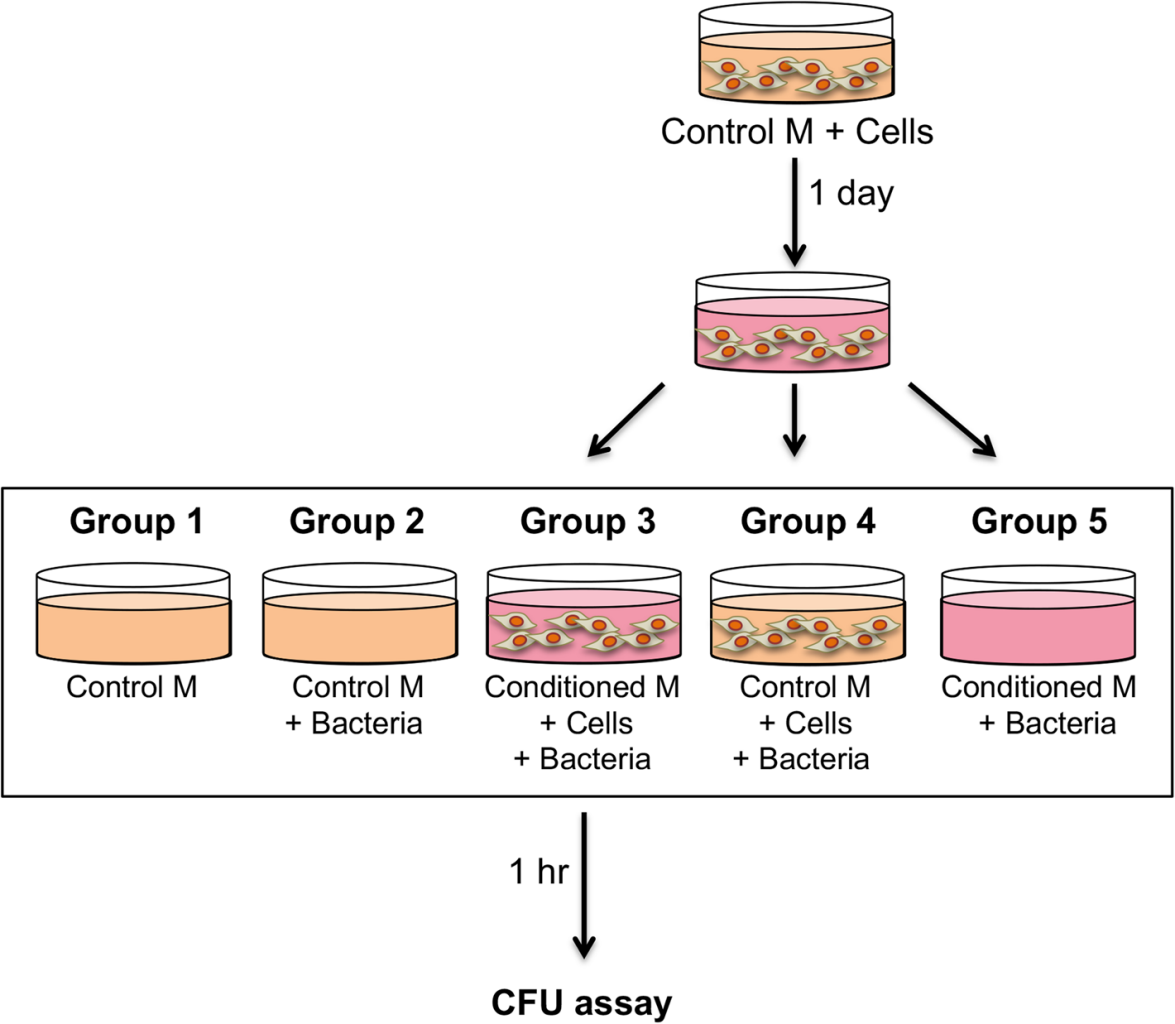
Experimental scheme. Analysis of the antibacterial activity of BM-MSCs and ASCs and cell-conditioned media. For details see the “Materials and methods.” M, medium

**Fig. 3:**
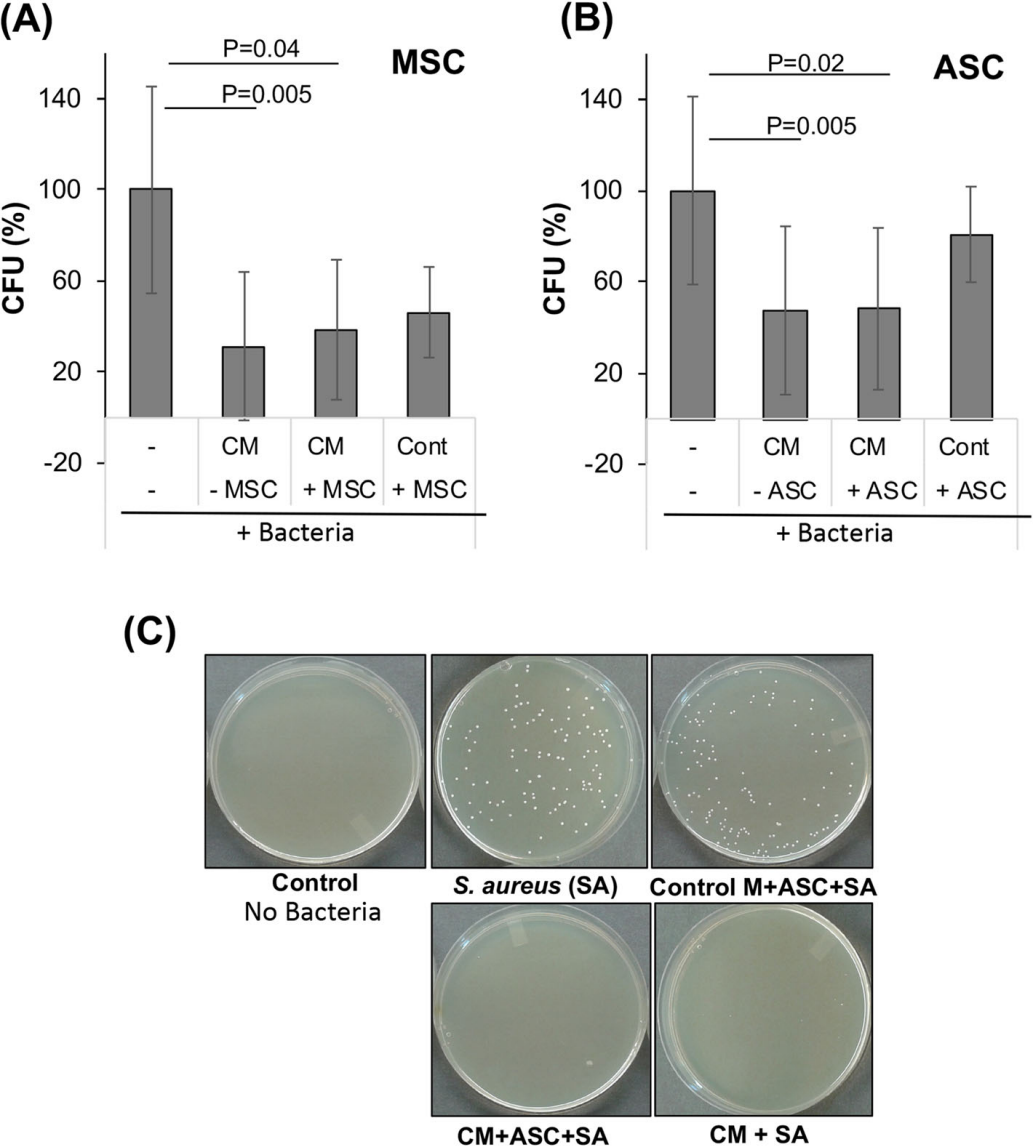
BM-MSCs and ASCs suppressed bacterial growth. Inhibition of bacterial growth upon exposure to **a** BM-MSCs and **b** ASCs. Cont, control medium; CM, conditioned medium. **c** Representative images of assay plates. BM-MSCs or ASCs were cultured in the growth medium, and three experimental groups inoculated with bacteria were prepared and analyzed: (1) culture medium alone, (2) conditioned medium from BM-MSCs or ASCs, (3) conditioned medium from BM-MSCs or ASCs containing cells, and (4) unconditioned culture medium containing BM-MSCs/ASCs. The total contents of each well (bacteria and medium) were plated for bacterial colony counting. CFU formation after 16–18 h were quantified, and the results compared to the bacteria control and expressed as percentages (mean + SD). For human ASCs, data are from 9 to 12 patients (*n* = 27 to 36), and for BM-MSCs, 2 patients (*n* = 12–21)

Next, to investigate this result in greater detail, experiments were performed to analyze the antibacterial activity of BM-MSCs and ASCs in cell-conditioned media (see experimental scheme in Fig. 2 and results in Fig. 3).

#### BM-MSCs

As shown in Fig. 3, no bacterial growth was observed in the control medium group, while the positive bacteria control showed 115 ± 50 CFU/plate (set as 100%), which was inhibited by ampicillin (Fig. 3a). Bacterial growth was significantly inhibited in BM-MSC-conditioned medium alone (31.9 ± 32.6%, *p* = 0.005 vs bacteria control; Fig. 3a) and in conditioned medium with BM-MSCs (38.6 ± 30.7%, *p* = 0.04 vs bacteria control; Fig. 3a). In comparison, in the control medium with BM-MSCs, bacterial growth was inhibited but not significantly (46.3 ± 19.8%; Fig. 3a).

#### ASCs

A similar analysis was performed with ASCs. No bacterial growth was observed in the control medium group. Positive bacteria control showed 114 ± 46.8 CFU/plate (set as 100%), which was inhibited by ampicillin (Fig. 3b, c). Bacterial growth was significantly inhibited in the conditioned medium (47.7 ± 36.9%, *p* = 0.005 vs bacteria control; Fig. 3b, c). Bacterial growth was also significantly inhibited in conditioned medium with ASCs (48.2 ± 35.5%, *p* = 0.02 vs bacteria control; Fig. 3b, c). In the control medium with ASCs, bacterial growth was inhibited somewhat but not significantly (80.8 ± 21%; Fig. 3b, c).

Overall, no significant differences were observed between ASCs and BM-MSCs.

### 1,25-Dihydroxy vitamin D_3_-treated ASCs elevated expression of antimicrobial protein LL-37

**Fig. 4:**
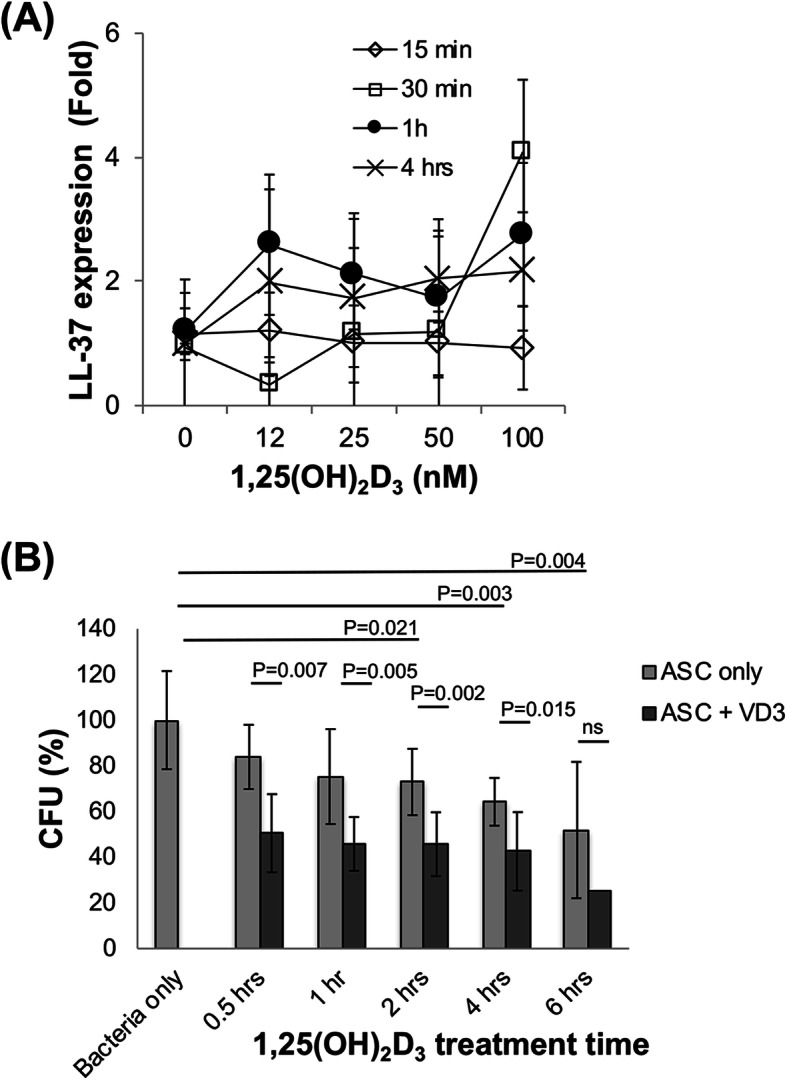
1,25-Hydroxy vitamin D_3_ treatment stimulated expression of LL-37 and enhanced antibacterial activity of ASCs. **a** LL-37 expression. ASCs cultured in the antibiotic-free medium were re-plated in tissue culture-treated 6-well plates (0.5 × 10^5^ cells/2 mL/well) and treated with a range of concentrations of 1,25(OH)_2_D_3_ (0 to 100 nM) for various time periods (0 to 4 h). At the end of the desired treatment time, the cultures were assayed for LL-37 gene expression by qRT-PCR. Results are expressed as fold increases compared to untreated cultures. Results are derived from 1 to 3 patients (*n* = 2 to 4) and expressed as mean ± SD. **b** Antibacterial activity. In another set of experiments, ASC cultures were treated with 100 nM 1,25(OH)_2_D_3_ for varying time periods, from 0.5 to 6 h, and the culture media were harvested and tested for antibacterial activity by CFU assay. With 1,25(OH)_2_D_3_ treatment, expression in ASCs was increased in a time- and dose-dependent manner; similarly, upon treatment with 100 nM 1,25(OH)_2_D_3_, ASC antibacterial activity was also enhanced as a function of time. Results are derived from 1 to 5 patients (*n* = 4 to 9) and expressed as mean ± SD, except ASCs exposed 1,25(OH)_2_D_3_ for 6 h (*n* = 1). ns: no significant. VD3: 1,25(OH)_2_D_3_

The main mechanism of antimicrobial action from MSCs was reported to be via the production of the cationic antimicrobial peptide, LL-37, which exerts multiple biological effects, including the prevention of biofilm formation, induction of immune mediators including IL-8, and regulation of the inflammatory response [47, 53]. In this study, to assess the potential enhancement of ASC antimicrobial activity, cells were treated with 1,25(OH)_2_D_3_, the active form of vitamin D_3_. It should be noted that recent reports showed that TLRs were stimulated by 1,25(OH)_2_D_3_ [50], reportedly resulting in higher LL-37 expression [48, 51]. Thus, cells were treated with a range of concentrations of 1,25(OH)_2_D_3_ (0 to 100 nM) for various time periods (15 min to 4 h). The results showed that treatment with 12.5 to 100 nM 1,25(OH)_2_D_3_ enhanced expression of LL-37 after 1 h (Fig. 4a). In comparison, BM-MSCs were also treated with 100 nM 1,25(OH)_2_D_3_ (0 to 100 nM) for various time periods (15 min to 4 h). The results showed that treatment with 1,25(OH)_2_D_3_ also enhanced the expression of LL-37 in BM-MSCs at 1 h (Supplemental Fig. 1a).

### 1,25(OH)_2_D_3_ stimulated ASCs showed enhanced antibacterial activity

We next examined the antibacterial activity of 1,25(OH)_2_D_3_-stimulated ASCs. Cells treated with 100 nM 1,25(OH)_2_ vitamin D_3_ for up to 6 h were tested in the CFU assay (bacteria control set as 100%; Fig. 4b). ASCs alone showed antibacterial activity in a time-dependent manner. ASCs exposed to 1,25(OH)_2_D_3_ further inhibited colony formation (CFU) as a function of time, from 30 min (ASCs: 83.9 ± 14.1%; ASCs + 1,25(OH)_2_D_3_: 50.5 ± 17.1%, *p* = 0.007 vs ASCs) until 4 h (ASCs: 64.3 ± 10.5%; ASCs + 1,25(OH)_2_D_3_: 42.6 ± 17.2%, *p* = 0.015 vs ASCs). For comparison, BM-MSCs were treated similarly and tested (see Supplemental Fig. 1b). Similarly, BM-MSCs alone showed antibacterial activity, and BM-MSCs exposed to 1,25(OH)_2_D_3_ further inhibited colony formation (CFU) as a function of time, from 30 min (BM-MSCs: 85.9%; BM-MSCs + 1,25(OH)_2_D_3_: 66.2%) until 6 h (BM-MSCs: 78.1%; BM-MSCs + 1,25(OH)_2_D_3_: 61.1%).

### Involvement of vitamin D and TLR receptors in the antibacterial activity of ASCs

**Fig. 5:**
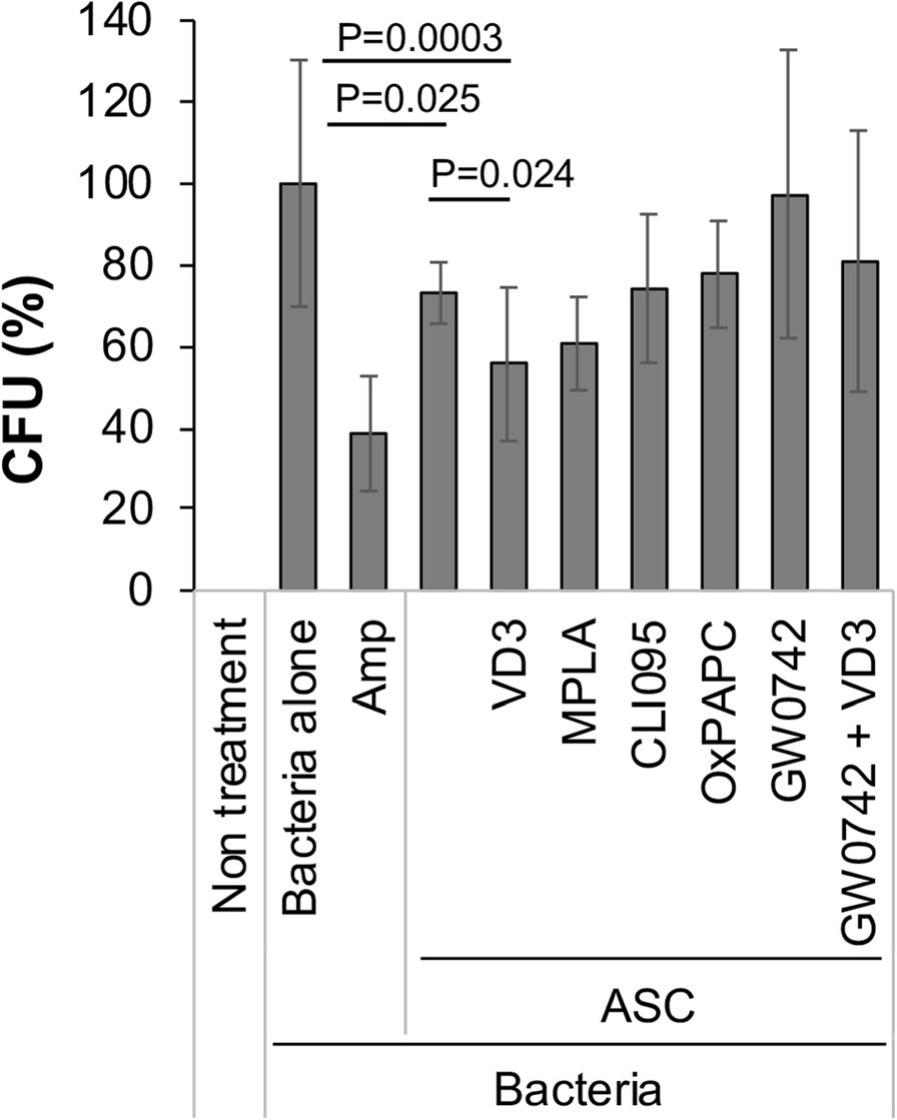
Involvement of vitamin D receptor in ASC antibacterial activity. ASCs cultured in antibiotic-free medium were replated in tissue culture-treated 6-well plates (0.5 × 10^5^ cells/2 mL/well) and treated for 1 day with the following: (1) TLR agonists, MPLA (12.5 μg/mL); TLR 4 antagonist, CLI-095 (2.5 μM), or TLR 2/4 antagonist, OXPAPC (25 μM) for 1 day; or (2) vitamin D receptor inhibitor, GW0742 (12.5 μM), with or without 1,25(OH)_2_D_3_ (100 nM). Antibacterial activity was assayed as described in the “Materials and methods.” The results showed that treatment with CLI-095, OxPAPC, and MPLA did not affect the antibacterial activity of ASCs. On the other hand, treatment with the vitamin D signaling inhibitor, GW0742, almost completely suppressed the antibacterial activity of 1,25(OH)_2_D_3_ activated and unactivated ASCs. Results are derived from 3 to 5 patients (*n* = 7 to 14) and expressed as mean ± SD. VD3: 1,25(OH)_2_D_3._

To investigate the subcellular biochemical mechanism of their antibacterial activity, ASCs were treated with the vitamin D receptor signaling inhibitor GW0742, and/or the TLR agonists (MPLA; monophosphoryl lipid A) or TLR4 antagonist CLI-095, or TLR2/4 antagonist OXPAPC, with or without 1,25(OH)_2_D_3_. The results are shown in Fig. 5 as a percent of bacterial colony formation (positive bacteria control set as 100%). As shown in Fig. 5, untreated ASCs alone yielded 73.1 ± 7.5 (*p* = 0.025 vs bacteria control) after 24 h incubation. In addition, 1,25(OH)_2_D_3_ treatment enhanced the antibacterial activity of ASCs (55.6 ± 18.8%, *p* = 0.0003 vs bacteria control). Treatment with CLI-095, OxPAPC, and MPLA did not affect the antibacterial activity of ASCs under this experimental condition. We also treated ASCs with the vitamin D signaling inhibitor, GW0742, which acts by blocking the interaction between vitamin D receptor and steroid receptor coactivator-2 (SRC-2), leading to the inhibition of the vitamin D signaling pathway. We observed that the antibacterial activity of 1,25(OH)_2_D_3_ activated as well as unactivated ASCs was almost completely suppressed by treatment with GW0742 (GW0742: 97.4 ± 35.4%, *p* = 0.867 vs bacteria control; GW0742 + 1,25-dihydroxy vitamin D_3_: 80.9 ± 32.1%, *p* = 0.204 vs bacteria control; Fig. 5).

## Discussion

Bones, which usually are well protected from infection, can become infected through three routes: (1) bloodstream, (2) direct invasion such as open fracture or surgery, and (3) direct infections from nearby structures such as joints or soft tissues. Infection can also spread outward from the bone to nearby joints and soft tissues. In fact, osteomyelitis commonly follows a similar infection process as a joint infection. In this study, we investigated the potential of adult mesenchymal stem cells, such as BM-MSCs and ASCs, as an adjunct antibacterial agent. In osteomyelitis, bone lesions with extensive osteolysis often require surgical debridement and removal of all infected tissues, resulting in significant tissue loss. Thus, for a cell-based therapeutic application, it would be ideal that the cells used also have regenerative or reparative activities. MSCs, which have strong osteogenic and regenerative activities, are naturally the preferred cell type—our study on MSCs that aims to show and enhance their antibacterial activity is therefore relevant for such application.

One method used in the past to determine the number of viable microbial cells in culture was based on quantitation of ATP, but with inconsistent results. Our lab actually pioneered the 16S RNA-based RT-PCR method for detecting joint infection [54] and recently adopted it for osteomyelitis [55]. However, this method detects all bacteria, including non-viable bacteria, potentially giving false positives. On the other hand, CFU assay is a direct, simple method that only counts viable bacteria (i.e., excluding dead bacteria and debris). This assay thus allows us to specifically assess the bactericidal activity of ASCs/BM-MSCs. We therefore chose CFU counting as a readily applicable and appropriate method for quantifying a viable bacterial number in this study.

We first investigated the antibacterial effect of BM-MSCs/ASCs in a simulated joint setting in vitro. In an assay mix containing synovial fluid and inoculated with bacteria, simulating septic arthritis, BM-MSCs/ASCs demonstrated antibacterial activity. It is noteworthy that, although MSCs/ASCs showed significant inhibition of bacteria growth in the presence of synovial fluid, their effects were lower compared to the group without synovial fluid (Fig. 1b). It is possible that some components of the synovial fluid, including hyaluronan, lubricin, proteinases, collagenase, and prostaglandins [56], could degrade, deactivate, or counteract against the secreted antimicrobial proteins by BM-MSCs and ASCs.

In this report, we have focused on human ASCs and demonstrated their antibacterial effects on *S. aureus* in vitro. Specifically, ASC-conditioned medium with or without additional cells suppressed bacterial growth (Figs. 1 and 3). On the other hand, bacteria incubated with ASCs in fresh control medium did not show any significant effect (Fig. 3a, b), suggesting that ASCs secreted antibacterial factors in growth medium during the 24-h incubation period. Also, it is noteworthy that our results showed that just 1-h incubation of bacteria with a conditioned medium is sufficient to suppress bacterial growth. In this study, ampicillin was used as a positive antibacterial control. It should be noted that we have purposely chosen to use an experimentally determined, low concentration of ampicillin that results in a mild, incomplete inhibition of bacteria growth, to allow room to measure the adjunct antibacterial activity of MSCs/ASCs. In this manner, the “ampicillin equivalence” of the antimicrobial activity of ASCs/BM-MSCs may be estimated, providing a therapeutic measure for future clinical applications.

We have also observed variable antibacterial activity in ASCs from different donors (data not shown), namely, ASCs from some donors almost completely blocked bacterial colony formation, while some others showed a relatively low level of activities, as indicated by the large standard deviations between results from different experiments throughout the study. It is possible that patients’ individual difference may greatly affect their antimicrobial activity. It is also possible that prior bacteria exposure could “prime” the ASCs/BM-MSCs to result in strong antimicrobial activity.

The main mechanism of antimicrobial action from MSCs has been reported to be via the production of the cationic antimicrobial peptide LL-37 [35]. We therefore tested the hypothesis that the antibacterial activity of ASCs may be mediated via LL-37. Our results showed that LL-37 was expressed in ASCs and that treatment of ASCs with 1,25(OH)_2_D_3_ elevated LL-37 expression level (Fig. 4a, b). However, we were unable to detect the level of LL-37 secreted from ASCs by using standard ELISA (data not shown), likely due to a low sensitivity of the commercially available ELISA kit.

LL-37 has a broad range of antibacterial activity against both Gram-negative and Gram-positive bacteria and has mostly been studied in vitro using the synthetic peptide [57–59]. The reported activities of LL-37 vary in several studies [58, 59], suggesting sensitivity to experimental conditions, such as salt, pH, and the phase of bacterial growth. In this study, we also tested the activity of LL-37 by directly treating the bacterial cultures with the LL-37 peptide. We found that higher concentrations of the LL-37 peptide (50% inhibitory concentration is approximately 10 μg/mL) showed inhibition of bacterial growth, but was ineffective at lower concentrations (data not shown). In comparison, our results showed significant bacterial inhibition in the presence of ASCs, suggesting the possibility of another type of antimicrobial factor, other than LL-37, particularly since treatment with TLR antagonists suppressed LL-37 expression but did not affect ASC antibacterial activity. Besides cathelicidin, antimicrobial peptides such as defensins, cystatin C, elafin, and lipocalin 2 are other potential candidates that could mediate this process as human antimicrobial peptides [60]. Recent studies also reported that the antibacterial activity of MSCs is mediated by secreted products found in MSC-conditioned medium [61, 62], which is also effective against biofilm formation [62, 63]. Our ongoing study aims to further identify the key factors responsible for the antibacterial activity of ASCs. According to the Antimicrobial Peptide Database (http://aps.unmc.edu/AP/main.php), 141 human host defense peptides have been registered in the database (as of June 2020). An extensive study of the antimicrobial peptide is necessary to find key factor(s) of antimicrobial activity in ASCs/BM-MSCs. Also, it has been reported that there is a synergism between LL-37 and other antimicrobial peptides in killing bacteria [64, 65]. In our preliminary study, human beta-defensin (HBD)-1 and HBD-2 expression were not detected in ASCs with or without 1,25(OH)_2_D_3_ treatment, whereas the expression of LL-37 was observed. It is still possible that LL-37 plays an important role in the combination with other antimicrobial peptides. Our study also showed no significant differences between ASCs and BM-MSCs. As our sample size was relatively small, increasing sample size would further test the significance level of our findings. Additional extensive analysis of the antimicrobial peptides produced by BM-MSCs and ASCs may reveal possible differences. In addition, a comparison of the antimicrobial activities of MSCs derived from different tissue sources which are processed under similar experimental conditions could also yield interesting information. Our results clearly showed that treatment with 1,25(OH)_2_D_3_ enhanced ASC antibacterial activity, in a time and dose-dependent manner. We plan to apply this information to test the therapeutic potential of MSCs/ASCs in a rabbit osteomyelitis model recently established in our laboratory (manuscript submitted for publication). We also observed that the antibacterial activity of ASCs was almost completely suppressed by the vitamin D receptor inhibitor, GW0742 (Fig. 5). Interestingly, our preliminary study showed that TLR4 expression did not change in ASCs with 1,25(OH)_2_D_3_ treatment (data not shown). These findings strongly suggest that vitamin D receptor plays a major role in ASC antibacterial activity. It is noteworthy that native ASCs alone, without 1,25(OH)_2_D_3_ treatment, showed significant antibacterial activity in a time-dependent manner, suggesting an endogenous, basal level of vitamin D signaling. It is noteworthy that MSCs may be activated to produce antimicrobial peptides by other biofactors related to inflammation (e.g., interleukin-1β [66]) and sepsis (e.g., bacterial lipopolysaccharides (LPS) [67]). However, given their known harmful bioactivities, the potential applicability of these agents for the treatment of osteomyelitis and related infections is severely limited. In contrast, 1,25(OH)_2_D_3_, shown here to enhance the antibacterial activity of ASCs, is also an actively pro-osteogenic factor for MSCs and promoter of bone growth [68] and should therefore be biocompatible for the treatment of bone-related infections.

Taken together, our findings reported here strongly suggest that adult human mesenchymal stem cells, including both BM-MSCs and ASCs, present as potential adjunct therapies in the treatment of orthopedic bacterial infections, such as septic arthritis and osteomyelitis. In addition, 1,25(OH)_2_D_3_, a natural metabolite, may significantly enhance the antibacterial activity and the therapeutic value of the cell-based therapy.

## Conclusion

Our findings clearly demonstrate that adult MSCs, i.e., ASCs and BM-MSCs, produce antibacterial biofactor(s) that inhibit the growth of *S. aureus* and that vitamin D plays a key regulatory role in this activity. The potential application of ASCs as a biocompatible, adjunct treatment of musculoskeletal infections, possibily augmented with the administration of 1,25(OH)_2_D_3_, needs to be assessed via in vivo testing in animal models of osteomyelitis.

## Supplementary information

**Additional file 1: Figure S1.** 1,25-dihydroxy vitamin D_3_ treatment stimulated expression of LL-37 and enhanced antibacterial activity in BM-MSCs.

## Data Availability

The data sets supporting the conclusion of this article are included within the article.

## Abbreviations

OM: Osteomyelitis
CFU: Colony-forming unit
MSC: Mesenchymal stem cell
ASC: Adipose-derived stem cell
MRSA: Methicillin-resistant *S. aureus*
MSSA: Methicillin-sensitive *S. aureus*
BM: Bone marrow
DMEM: Dulbecco’s modified Eagle’s medium
MEM: α-Minimum Essential Medium
Anti-anti: Antibiotic-antimycotic
FBS: Fetal bovine serum
PBS: Phosphate-buffered saline
FGF-2: Fibroblast growth factor-2
Amp: Ampicillin
IRB: Institutional Review Board
IPFP: Infrapatellar fat pad
TSB: Tryptic soy broth
MPLA: Monophosphoryl lipid A
TLR: Toll-like receptor
LPS: Lipopolysaccharides
SRC-2: Steroid receptor coactivator-2
