# Supplementary material for: Antimicrobial activity of mesenchymal stem cells against Staphylococcus aureus
Source: Stem Cell Res Ther. 2020 Jul 17;11:293. doi: 10.1186/s13287-020-01807-3 (PMC7367313; doi:10.1186/s13287-020-01807-3)
Supplement: Supplementary file 1 — Additional file 1: Figure S1. 1,25-dihydroxy vitamin D3 treatment stimulated expression of LL-37 and enhanced antibacterial activity in BM-MSCs. [file 13287_2020_1807_MOESM1_ESM.zip › SCRT-D-20-00500_Suppl Figure 1.Legend.docx]

**Supplemental Figure Legend**

**Figure 1. 1,25-dihydroxy vitamin D_3_ treatment stimulated expression of LL-37 and enhanced antibacterial activity in BM-MSCs.** (A) LL-37 expression. BM-MSCs cultured in antibiotic-free medium were re-plated in tissue culture treated 6-well plates (0.5 × 10^5^ cells/2 mL/well) and treated with a range of concentrations of 1,25(OH)_2_D_3_ (0 to 100 nM) for various time periods (0 to 4 hours). At the end of each treatment time, the cultures were assayed for LL-37 gene expression by qRT-PCR. Results are expressed as fold increases compared to untreated cultures. Results are derived from1 patient (n = 1). (B) Antibacterial activity. In another set of experiments, BM-MSC cultures were treated with 100 nM 1,25(OH)_2_D_3_ for up to 6 hours, and the culture media were harvested and tested for antibacterial activity by CFU assay. With 1,25(OH)_2_D_3_ treatment, expression in BM-MSCs was increased, upon treatment with 100 nM 1,25(OH)_2_D_3_, BM MSC antibacterial activity was also enhanced. Results are derived from 1 patient (n = 1). h indicates hours. VD3: 1,25(OH)_2_D_3._
