# Supplementary figures and images for: Antimicrobial activity of mesenchymal stem cells against Staphylococcus aureus
Source: Stem Cell Res Ther. 2020 Jul 17;11:293. doi: 10.1186/s13287-020-01807-3 (PMC7367313; doi:10.1186/s13287-020-01807-3)

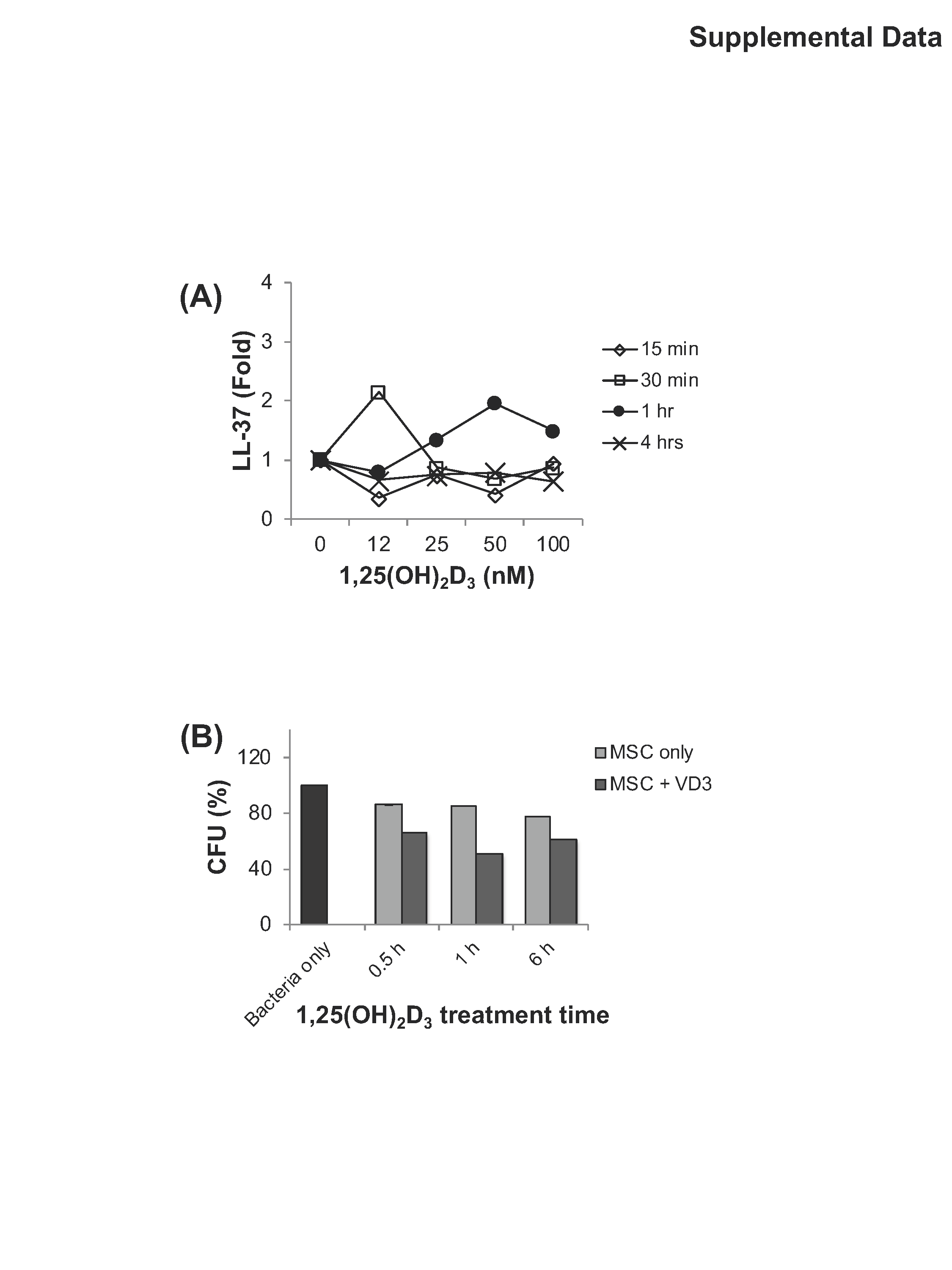

Supplement: Supplementary file 1 — Additional file 1: Figure S1. 1,25-dihydroxy vitamin D3 treatment stimulated expression of LL-37 and enhanced antibacterial activity in BM-MSCs. [file 13287_2020_1807_MOESM1_ESM.zip › SCRT-D_20-00500-Supplemental data_HY.tiff]
